# Effectiveness of community outreach and engagement in recruitment success for a prebirth cohort

**DOI:** 10.1017/cts.2017.7

**Published:** 2017-07-19

**Authors:** Beth B. Tigges, Jill L. Kaar, Nancy Erbstein, Pamela Silberman, Kate Winseck, Maria Lopez-Class, Thomas M. Burbacher

**Affiliations:** 1 University of New Mexico College of Nursing, Albuquerque, NM, USA; 2 Department of Pediatrics, University of Colorado School of Medicine, Aurora, CO, USA; 3 Department of Human Ecology, University of California-Davis, Davis, CA, USA; 4 International Rescue Committee, Salt Lake City, UT, USA; 5 Office of Disease Prevention, Office of the Director, National Institutes of Health, Bethesda, MD, USA; 6 *Eunice Kennedy Shriver* National Institute of Child Health and Human Development, National Institutes of Health, Bethesda, MD, USA; 7 Department of Environmental and Occupational Health Sciences, University of Washington School of Public Health, Seattle, WA, USA

**Keywords:** community outreach, community engagement, recruitment, prebirth cohort

## Abstract

**Introduction:**

We describe the effectiveness of community outreach and engagement in supporting recruitment for the US National Children’s Vanguard Study between 2009 and 2012.

**Methods:**

Thirty-seven study locations used 1 of 4 strategies to recruit 18–49-year-old pregnant or trying to conceive women: (1) Initial Vanguard Study used household-based recruitment; (2) Direct Outreach emphasized self-referral; (3) Enhanced Household-Based Recruitment enhanced Initial Vanguard Study strategies; and (4) Provider-Based Recruitment recruited through healthcare providers. Outreach and engagement included advance letters, interactions with healthcare providers, participation in community events, contacts with community organizations, and media outreach.

**Results:**

After 1–2 years, 41%–74% of 9844 study-eligible women had heard about the National Children’s Vanguard Study when first approached. Women who heard were 1.5–3 times more likely to consent. Hearing via word-of-mouth or the media most frequently predicted consent. The more sources women heard from the higher the odds of consent.

**Conclusions:**

We conclude that tailored outreach and engagement facilitate recruitment in cohort studies.

## Introduction

Recruitment of participants into longitudinal, observational studies that do not include any direct health benefit is a challenge [1], yet such studies are critical to identifying the incidence and natural history of many pediatric health conditions. There is a growing body of evidence regarding recruitment strategies for randomized clinical trials [2, 3], yet few evidence-based recommendations regarding effective recruitment in cohort studies. Community outreach and engagement is one commonly identified tactic [4–6]. Reviews of community-based research suggest community engagement may increase enrollment [7]. The literature contains reports of outreach and engagement as a passive recruitment strategy whereby study information is made widely available throughout the community to encourage potential participants to contact study recruiters [8–11]. Little is known about the effectiveness of outreach and engagement in a preparatory role for active recruitment in which potential participants are contacted by staff and invited to join a study. From 2009 to 2012, the National Children’s Study (NCS) Vanguard Study implemented outreach and engagement in support of both passive and active recruitment across the United States. In this article, we describe the effectiveness of those strategies in reaching eligible women and in supporting consent.

### The NCS Vanguard Study

The Vanguard Study was a pilot study that enrolled women and their children born in 37 diverse US locations (counties or parts of counties) to evaluate the feasibility, acceptability, and cost of recruitment and operations for the NCS, a large-scale epidemiological cohort study of children and their parents [12, 13]. Data were to be collected from prepregnancy until the children reached adulthood. Ultimately, it was decided that moving forward with the NCS was not feasible as designed. The Vanguard Study was closed in December 2014 [13].

The recruitment phase of the Vanguard Study began in 2009 with the Initial Vanguard Study (IVS) (see Table 1) [14]. The IVS was conducted at 7 locations; field workers enumerated households in predetermined geographical areas to identify and consent eligible women. Based on early results, a decision was made to evaluate additional recruitment strategies in an Alternate Recruitment Substudy (ARS), which began November 2010. The ARS evaluated three different recruitment strategies in 30 additional locations: Direct Outreach (DO) [15], Enhanced Household-Based Recruitment (EHBR) [16], and Provider-Based Recruitment (PBR) [17]. The DO strategy recruited women using community outreach and engagement, including direct mailing. Interested individuals contacted study centers and were screened via an in-person or phone interview, or a self-administered questionnaire. The EHBR strategy used improved staff training and more targeted outreach and engagement to enhance the household enumeration strategy of the IVS. The PBR strategy worked with healthcare providers to identify women of childbearing age for eligibility screening and recruitment.

**Table 1:** Overview of National Children’s Vanguard Study (NCS) recruitment groups and outreach and engagement approaches

Recruitment group	Number of study locations	Recruitment methods	Outreach and engagement approaches
Initial Vanguard Study (IVS)(January 2009–February 2012)	7	Household-based enumeration and recruitment	Community Advisory Boards Advance letters Healthcare provider (secondary) Community events Community organizations Media outreach (local)
Alternate Recruitment Substudy			
Direct Outreach (DO)(November 2010–February 2012)	10	Self-referral after widespread community outreach and engagement	Pre-study outreach and engagement plans Community Advisory Boards Advance letters with follow-up mailings Healthcare provider (secondary) Community events Community organizations Media outreach (national and local)—included television, radio, online and social media
Enhanced Household-Based Recruitment (EHBR) (November 2010–February 2012)	10	Enhanced household-based enumeration and recruitment Enhancements: (1) Improved staff training for enumeration and screening (2) Enhanced outreach and engagement	Pre-study outreach and engagement plans Community Advisory Boards Advance letters with follow-up mailings Healthcare provider (secondary) Community events Community organizations Media outreach (national and local)—included television, radio, online and social media
Provider-Based Recruitment (PBR) (November 2010–February 2012)	10	Recruitment via healthcare providers’ offices	Pre-study outreach and engagement plans Community Advisory Boards Advance letters with follow-up mailings Healthcare provider (primary) Community events Community organizations Media outreach (national and local)—included television, radio, online and social media

### NCS Outreach and Engagement

Recruitment at each location was conducted by a study center. Study centers were universities or research organizations, similar to academic investigative sites, under contract with the National Institutes of Health (NIH) to conduct the day-to-day operations of the study. Some centers covered more than 1 location. Each center was charged with implementing outreach and engagement to support recruitment. Although some supporting materials were centrally developed by NIH (eg, logos, web sites, pamphlets, posters, small giveaways), centers generally tailored methods and materials for localities. Outreach and engagement strategies were conducted for up to 2 years before the start of recruitment. All study centers hired local staff and had Community Advisory Boards to recommend best approaches to tailoring materials and activities. Most centers individualized the NCS logo for their location and ordered NCS-branded gear and identification badges.

Strategies generally fell into 5 categories: (1) advance letters; (2) healthcare providers; (3) community events; (4) community organizations; and (5) media (for data analysis, these categories were later divided into more specific groups of sources as reported by study participants: advance letters, healthcare providers, family/friends, other person, community organizations, and media). Except for 3 PBR locations, all centers mailed advance letters to households in predetermined geographical areas telling them about the study. Centers also conducted in-person or mailed outreach and engagement with local healthcare providers to inform them about the study, encourage them to either participate or recommend participants. All centers participated in community events (eg, local fairs, kindergarten registration) to distribute NCS information and giveaways (eg, water bottles) and, in the ARS, conduct supplemental recruitment. Centers also visited community organizations and businesses (eg, neighborhood associations, grocery stores) to raise study awareness, gain support from key formal and informal community leaders, and ask for suggestions for improving recruitment.

Media outreach efforts were both nationally and locally produced and included television (TV), radio, billboards, print, online, and social media. The NIH NCS Program Office implemented time-limited national media buys for TV, radio, and billboards in ARS locations, supplied centrally produced pamphlets and posters, and hosted a national web site with linked individualized study center sites. Centers varied in how extensive their tailored local campaigns were. All centers developed their own tailored local print materials; many placed ads in local newspapers, made regular press releases, or included mailings in local utility bills. Some ARS centers also purchased online advertisements and maintained Facebook pages, active Twitter feeds, and NCS blogs. In general, ARS locations had more widespread local campaigns than IVS locations, particularly with respect to TV, radio, online, and social media.

## Materials and Methods

### Sampling Frame

The original sample design for the NCS was a nationally representative, multistage, area probability sample, designed to provide estimates that could be generalized to the US population with subsets of the primary sampling units, generally equivalent to counties, selected to serve as locations; however, the 37 pilot locations of the NCS Vanguard were chosen to pretest field operations and not selected randomly. Some attempt at balancing demographic characteristics was made, but without statistical precision.

The second sampling stage was the selection of geographical segments within the primary sampling units. Segments were based on aggregations of contiguous census blocks with the measure of size being estimated annual births during the enrollment period. In combination, these secondary sampling units would yield ~250 births per year per primary sampling unit. The study centers consulted with local community representatives to get input on potential segment boundaries, so the segments would reflect coherent neighborhood groups to facilitate community outreach.

### Sample

**Fig. 1:**
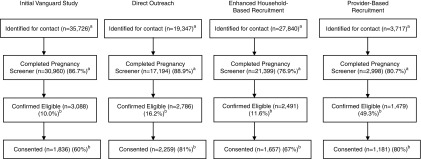
Flow diagram of samples by type of recruitment. ^a^Source: National Children’s Study [18]. ^b^Sample for this study. IVC Participant Recruitment Dataset V2.1, IVC Enrolled Women Analysis File V2.1, IVC Recruitment Analysis File V2.1, and ARS Analysis File V3.1.

Recruitment consisted of 2 stages. First, women in the secondary sampling units who were identified by household enumeration (IVS, EHBR), self-referral (DO), or provider referral (PBR) and were 18–49 years old were asked to complete a study eligibility questionnaire, the Pregnancy Screener (see Fig. 1). Second, those women who were pregnant or actively trying to get pregnant were invited to participate. In the IVS, pregnant minors (<18 years old) were eligible if the state they resided in defined them as emancipated; minors were ineligible in the 3 ARS groups. Women who were older than 49 and pregnant were eligible in both the IVS and ARS.

### Procedures

The study protocol was approved by the *Eunice Kennedy Shriver* National Institute of Child Health and Human Development Institutional Review Board (IRB) as the national federated IRB [19] or, depending on study center choice, a local IRB. A waiver of written documentation of consent allowed staff to obtain verbal informed consent from women for completion of the Pregnancy Screener. Women from the IVS, EHBR, and PRB locations were interviewed by trained interviewers or computer-assisted personal interviewing; women in the DO locations were either interviewed or completed a self-administered Pregnancy Screener and returned it via mail. The Pregnancy Screener was available in English and Spanish. Spanish-speaking interviewers interviewed Spanish-speaking women. If women’s primary spoken language was other than Spanish, interviews were conducted in the women’s language whenever possible.

### Measures

This analysis employs 7 self-reported demographic items and 2 items about familiarity with the NCS from the initial enumeration questionnaire used in the IVS and Pregnancy Screener, used in both the IVS and ARS. The demographic items included age, race, ethnicity, primary language, marital status (married; not married—living with partner or never married; separated, divorced, or widowed), highest level of education completed (<high school diploma or general educational development; high school diploma or general educational development; some college/associate degree; bachelor’s degree; postgraduate degree), and annual family income (<$30,000; $30–$49,999; $50–$99,999; >$100,000). Education and income data were not available for the IVS. A composite race/ethnicity variable was created and included 5 groups: Hispanic; non-Hispanic White, African-American, Asian, and other. The first of the items about NCS familiarity was “Before today, had you heard about the National Children’s Study?” Response choices were: yes, no, refused, and don’t know. Those participants who responded “yes” to the first question were then asked “How did you hear about the National Children’s Study?” There were 13 possible response options for the IVS and 23 for the ARS, including options such as advance letter, healthcare provider, family member, the Special Supplemental Nutrition Program for Women, Infants, and Children (WIC) or another social agency, TV, and other. Respondents were encouraged to list all the ways that they had heard about the NCS.

### Data Analysis

All data were submitted electronically by study centers to the contractors identified by the NIH Program Office and were analyzed by another private contractor using SAS/STAT^TM^ software. This analysis used the IVC Participant Recruitment Dataset V2.1, IVC Enrolled Women Analysis File V2.1, IVC Recruitment Analysis File V2.1, and ARS Analysis File V3.1 (based on April 11, 2014 Virtual Data Repository submission). Data files used were not final versions and data presented may be slightly different from other published versions. Differences in demographic variables between recruitment groups were analyzed using χ^2^ for categorical variables and the Kruskal-Wallis for the non-normal, continuous age variable. Logistic regression was used to analyze prediction models of hearing about the NCS and consenting for the NCS. Observations that contained missing values for either the dependent or independent variables were excluded from the regression.

## Results

Our results describe the effectiveness of outreach and engagement in helping eligible women hear about the study and in promoting their consent. We address 6 research questions: (1) Did community outreach and engagement result in women having heard about the NCS when first contacted? (2) Did more women hear about the NCS in the ARS than the IVS? (3) How did women hear about the NCS? (4) Were women who had heard about the NCS more likely to consent? (5) Were certain sources of hearing about the NCS or the number of sources related to consent? (6) Were all demographic sub-populations equally likely to have heard about the NCS?

The 9844 women who completed the Pregnancy Screener and were confirmed eligible for consent are the sample for this article (see Fig. 1): 3088 from the IVS; 2786 from the DO; 2491 from the EHBR; and 1479 from the PBR recruitment groups. Table 2 displays the sample characteristics. There were significant differences between recruitment groups in all demographic characteristics (χ^2^=169.4−1180.9, *P*<0.001). However, because this was a feasibility study and the duration of participant recruitment varied across and within recruitment groups, Table 2 data are presented for descriptive purposes only, not as a basis for comparison across ARS strategies. Details about demographic characteristics of samples compared with local populations have been presented elsewhere [14–17].

**Table 2:** Demographic characteristics of women eligible for consent for the National Children’s Study

	Sample									
			Alternate Recruitment Substudy							
Demographic characteristics	IVS* (n=3088)		DO (n=2786)		EHBR (n=2491)		PBR (n=1479)		Total (n=9844)	
Age [Mean (SD)]	28.62 (5.64)		30.06 (5.88)		29.13 (6.52)		28.00 (6.33)		29.07 (6.08)	
	n	%	n	%	n	%	n	%	n	%
Race/ethnicity										
Non-Hispanic White	1457	53.04	2007	72.53	1289	52.38	712	52.51	5465	58.57
Hispanic	479	17.44	246	8.89	572	23.24	204	15.04	1501	16.09
Non-Hispanic African-American	158	5.75	312	11.28	279	11.34	357	26.33	1106	11.85
Non-Hispanic Asian	157	5.72	112	4.05	112	4.55	26	1.92	407	4.36
Non-Hispanic other	496	18.06	90	3.25	209	8.49	57	4.20	852	9.13
Language										
English	2552	91.73	2625	96.12	1922	85.23	1116	91.40	8215	91.39
Spanish	209	7.51	74	2.71	289	12.82	93	7.62	665	7.40
Other	21	0.75	32	1.17	44	1.95	12	0.98	109	1.21
Marital status										
Married	2081	71.66	2194	79.18	1514	61.39	677	47.88	6466	67.67
Not married (living with partner or never married)	683	23.52	522	18.84	857	34.75	673	47.60	2735	28.62
Separated, divorced, or widowed	140	4.82	55	1.98	95	3.85	64	4.53	354	3.70
Education										
<High school or GED			142	5.14	416	16.94	237	16.83	914	12.35
High school or GED			320	11.58	568	23.13	354	25.14	1357	18.34
Some college/associate degree			769	27.83	759	30.90	444	31.53	2192	29.63
Bachelors			936	33.88	451	18.36	210	14.91	1809	24.45
Postgraduate			596	21.57	262	10.67	163	11.58	1127	15.23
Income										
<$30,000			831	30.44	787	37.64	667	49.59	2285	37.06
$30,000–$49,999			536	19.63	422	20.18	231	17.17	1189	19.28
$50,000–$99,999			860	31.50	629	30.08	293	21.78	1782	28.90
>$100,000			503	18.42	253	12.10	154	11.45	910	14.76

IVS, Initial Vanguard Study; DO, Direct Outreach; EHBR, Enhanced Household-Based Recruitment; PBR, Provider-Based Recruitment; GED, general educational development.

* Including women from both the original IVS period of January 2009 to September 2010 and beyond.

### How Women Heard About the Study

Overall, most women who were study eligible had already heard about the NCS before their first contact with recruiters (Table 3). ARS women had heard more than IVS women. Advance letters, media outreach, and friends, family, or other people were the most frequent ways that IVS, DO, and EHBR women had heard about the study before being approached by data collectors. Healthcare providers, as expected, and media were the most frequent for the PBR women. Community organizations were one of the least frequent ways women had heard across all groups. Most women reported that they had heard about the study from only 1 source.

**Table 3:** Hearing about the National Children’s Study (NCS) among women eligible for consent

	Sample									
			Alternate Recruitment Substudy							
	IVS (n=3060)		DO (n=2775)		EHBR (n=2452)		PBR (n=1365)		Total (n=9652)	
	n	%	n	%	n	%	n	%	n	%
Heard about the NCS										
Yes	1258	41.1	2065	74.4	1319	53.8	860	63.0	5502	57.0
How heard*										
Advance letter	642	51.0	853	41.3	470	35.6	149	17.3	2114	38.4
Healthcare provider†	84	6.7	168	8.1	102	7.7	489	56.9	843	15.3
Friends/family‡	225	17.9	248	12.0	111	8.4	62	7.2	646	11.7
Other person§	69	5.5	272	13.2	148	11.2	63	7.3	552	10.0
Community organization‖	39	3.1	222	10.8	60	4.6	53	6.2	374	6.8
Media¶	264	21.0	872	42.2	401	30.4	171	19.9	1708	31.0
Number of sources										
1	825	65.6	1256	60.8	850	64.4	584	67.9	3515	63.9
2	200	15.9	463	22.4	173	13.1	151	17.6	987	17.9
>3	32	2.5	135	6.5	31	2.4	27	3.7	230	4.2
No response	201	16.0	211	10.2	265	20.1	93	10.8	770	14.0

IVS, Initial Vanguard Study; DO, Direct Outreach; EHBR, Enhanced Household-Based Recruitment; PBR, Provider-Based Recruitment.

* Participants may select more than 1 source, so column totals may exceed 100%.

† IVS: doctor or healthcare provider; Alternate Recruitment Substudy (ARS): prenatal care provider or other healthcare provider.

‡ IVS: friends or family; ARS: other friend or family member.

§ IVS: someone else in community or community leader; ARS: other NCS participant, neighbor, co-worker, community partners, outreach event.

‖ IVS: Church, synagogue, other religious; ARS: school, WIC, other social agency, or religious organization.

¶ IVS: billboards, newspaper, television, or radio; ARS: print media, television, or radio.

### Community Outreach and Engagement and Women’s Consent

After adjusting for demographics, women who had heard about the NCS (yes/no) were an estimated 1.5–3 times more likely to consent to participate than women who had not [IVS: odds ratio (OR)=1.67, 95% confidence interval (CI): 1.40, 1.98; DO: OR=2.90, 95% CI: 2.35, 3.59; EHBR: OR=1.58, 95% CI: 1.29, 1.94; PBR: OR=2.30, 95% CI: 1.61, 3.26]. This effect was strongest for DO and PBR, and weakest for IVS and EHBR. Among women who had heard, the importance of how women heard varied across recruitment groups after controlling for demographics (Table 4). In the IVS, no one category of outreach and engagement was associated with consents. In the ARS, the advance letter was not associated with DO or EHBR women’s consent. However, PBR women who heard about the study by advance letter were more likely to consent than those who heard only from other sources. Likewise, DO and EHBR women who heard via the media were more likely to consent. EHBR women who heard from a healthcare provider, friends, or family were more likely to consent. And DO and PBR women who heard from another person were more likely to consent than women who had not. Across all recruitment groups, the more sources women heard from, the greater the odds of consent, with a slight variation in the PBR group (Table 5).

**Table 4:** Logistic regression of type of outreach on consent status among women who had heard about the National Children’s Study (NCS) (by recruitment type, controlling for demographic variables)

	Recruitment type							
	IVS (n=1039)		DO (n=1995)		EHBR (n=1089)		PBR (n=712)	
	OR	95% CI	OR	95% CI	OR	95% CI	OR	95% CI
How heard about the NCS*								
Advance letter	1.21	0.91, 1.61	0.85	0.64, 1.14	1.18	0.87, 1.61	**2**.**52**	**1.14**, **5.57**
Healthcare provider†	1.36	0.77, 2.43	1.17	0.69, 1.98	**2**.**62**	**1.38**, **4.95**	1.32	0.77, 2.24
Friends/family‡	1.16	0.80, 1.67	1.47	0.91, 2.38	**2**.**49**	**1.35**, **4.60**	0.97	0.37, 2.53
Other person§	1.61	0.87, 2.98	**1**.**65**	**1.05**, **2.61**	1.20	0.76, 1.89	**8**.**92**	**1.19**, **67.04**
Community organization‖	1.31	0.59, 2.91	1.60	0.95, 2.67	1.90	0.87, 4.15	2.72	0.79, 9.39
Media¶	1.16	0.84, 1.62	**1**.**47**	**1.10**, **1.98**	**1**.**57**	**1.14**, **2.15**	0.94	0.52, 1.72

IVS, Initial Vanguard Study; DO, Direct Outreach; EHBR, Enhanced Household-Based Recruitment; PBR, Provider-Based Recruitment; OR, odds ratio; CI, confidence interval. Bold value indicates that CI does not contain 1.00; P-value for the Hosmer and Lemeshow goodness-of-fit test: IVS=0.14; DO=0.96; EHBR=0.16; PBR=0.68.

* Reference category for each individual source is not hearing from that source.

† IVS: doctor or healthcare provider; Alternate Recruitment Substudy (ARS): prenatal care provider or other healthcare provider.

‡ IVS: friends or family; ARS: other friend or family member.

§ IVS: someone else in community or community leader; ARS: other NCS participant, neighbor, co-worker, community partners, outreach event.

‖ IVS: Church, synagogue, other religious; ARS: school, WIC, other social agency, or religious organization.

¶ IVS: billboards, newspaper, TV, or radio; ARS: print media, TV, or radio.

**Table 5:** Logistic regression of number of sources heard about National Children’s Study (NCS) on consent status among women eligible for consent (by recruitment type, controlling for demographic variables)

	Recruitment type							
	IVS (n=2313)		DO (n=2476)		EHBR (n=1693)		PBR (n=977)	
	OR	95% CI	OR	95% CI	OR	95% CI	OR	95% CI
Number of sources*,†								
1	**1**.**60**	**1.32**, **1.95**	**2**.**62**	**2.08**, **3.30**	**1**.**58**	**1.26**, **1.98**	**1**.**82**	**1.25**, **2.63**
2	**2**.**00**	**1.41**, **2.83**	**4**.**18**	**2.93**, **5.95**	**2**.**39**	**1.54**, **3.70**	**6**.**08**	**2.54**, **14.57**
3–6‡	**6**.**24**	**1.87**, **20.87**	**4**.**41**	**2.41**, **8.06**	**8**.**94**	**2.09**, **38.20**	4.36	0.99, 19.17

IVS, Initial Vanguard Study; DO, Direct Outreach; EHBR, Enhanced Household-Based Recruitment; PBR, Provider-Based Recruitment; OR, odds ratio; CI, confidence interval. Bold value indicates that CI does not contain 1.00; *P*-value for the Hosmer and Lemeshow goodness-of-fit test: IVS=0.95; DO=0.76; EHBR=0.04; PBR=0.23.

* Reference category is not hearing from any source.

† Six possible sources: advance letter, healthcare provider, friends/family, other person, community organization, media (see Table 3 for operational definitions).

‡ Categories 3–6 were combined to provide stable OR estimates.

### Demographics and Hearing About the Study

Table 6 presents the logistic regression to assess whether demographic variables predicted whether a woman reported that she had heard about the NCS. Because of the many significant interactions between all demographic variables and recruitment type, separate logistic regression models were built for each recruitment group. In general, across all recruitment groups, women who were Hispanic, African-American, Asian, or another non-Hispanic race/ethnicity were much less likely to have heard about the NCS than non-Hispanic White women, with a few significant exceptions. There were no differences in hearing about the NCS between EHBR non-Hispanic White and Hispanic women, the group with the highest percentage (23%) of Hispanics. And DO African-American women were almost twice as likely to have heard about the NCS as non-Hispanic Whites.

**Table 6:** Logistic regression of demographic variables on hearing about the National Children’s Study (NCS) among women eligible for consent by recruitment type

	Recruitment type							
	IVS (n=2470)		DO (n=2668)		EHBR (n=1891)		PBR (n=1051)	
Demographic characteristics	OR	95% CI	OR	95% CI	OR	95% CI	OR	95% CI
Age								
Raw	1.02	1.00, 1.03	0.98	0.97, 1.00	1.02	1.00, 1.03	1.02	0.99, 1.05
Race/ethnicity								
Non-Hispanic White*								
Hispanic	**0**.**52**	**0.39**, **0.70**	**0**.**68**	**0.48**, **0.96**	0.92	0.68, 1.25	**0**.**28**	**0.17**, **0.46**
Non-Hispanic African-American	**0**.**53**	**0.35**, **0.80**	**1**.**95**	**1.32**, **2.87**	**0**.**42**	**0.30**, **0.60**	**0**.**31**	**0.22**, **0.44**
Non-Hispanic Asian	**0**.**23**	**0.15**, **0.35**	**0**.**34**	**0.21**, **0.54**	**0**.**59**	**0.35**, **0.99**	**0**.**30**	**0.09**, **0.96**
Non-Hispanic other	**0**.**80**	**0.64**, **0.99**	0.88	0.53, 1.45	**0**.**65**	**0.45**, **0.94**	0.53	0.25, 1.11
Language								
English*								
Non-English	0.69	0.45, 1.05	1.18	0.71, 1.97	0.71	0.49, 1.02	1.58	0.84, 2.97
Marital status								
Married*								
Not married (living with partner or never married)	**0**.**53**	**0.42**, **0.66**	**0**.**66**	**0.50**, **0.88**	0.87	0.68, 1.11	0.92	0.64, 1.31
Separated, divorced, or widowed	0.82	0.56, 1.20	0.59	0.32, 1.10	0.72	0.43, 1.20	1.43	0.70, 2.90
Education								
<High school or GED			1.55	0.92, 2.60	**0**.**65**	**0.46**, **0.91**	1.16	0.77, 1.77
High school or GED*								
Some college/associate degree			1.13	0.82,1.55	1.22	0.94, 1.59	1.21	0.85, 1.71
Bachelors			**1**.**50**	**1.07**, **2.11**	**1**.**56**	**1.11**, **2.17**	1.31	0.74, 2.34
Postgraduate			**1**.**80**	**1.23**, **2.64**	**2**.**27**	**1.51**, **3.42**	**2**.**68**	**1.25**, **5.75**
Income								
<$30,000			1.00	0.75, 1.33	1.07	0.80, 1.43	1.02	0.69, 1.50
$30,000–$49,999*								
$50,000–$99,999			**0**.**73**	**0.55**, **0.95**	0.90	0.67, 1.20	1.39	0.82, 2.36
>$100,000			**0**.**53**	**0.38**, **0.72**	0.89	0.61, 1.31	1.33	0.64, 2.73

IVS, Initial Vanguard Study; DO, Direct Outreach; EHBR, Enhanced Household-Based Recruitment; PBR, Provider-Based Recruitment; OR, odds ratio; CI, confidence interval; GED, general educational development. Bold value indicates that CI does not contain 1.00; *P*-value for the Hosmer and Lemeshow goodness-of-fit test: IVS=0.72; DO=0.74; EHBR=0.32; PBR=0.54.

* Referent category for logistic regression.

Age and primary language spoken at home were not associated with hearing about the NCS for any of the recruitment groups. In the EHBR and PBR groups, women who were not married were as likely to report hearing about the NCS as women who were married. In contrast, IVS and DO unmarried women were less likely than married women to have heard. Education and income data were not collected for the IVS group. However, across all 3 ARS groups, women who had bachelors or graduate degrees were more likely to have heard than high school graduates. DO women with annual incomes of $50,000 or higher were less likely than women with incomes of $30–$49,999 to report hearing about the study. Income was not associated with hearing about the NCS in the other 2 ARS groups.

## Discussion

The NCS is the one of the first studies to demonstrate that, while controlling for demographic variables, effective outreach and engagement predicts more successful cohort recruitment. Effective outreach and engagement was measured by whether women had heard about the NCS. Women who had heard before having contact with recruiters were more likely to consent. Consents increased both when outreach and engagement were used as a passive recruitment strategy and women needed to self-refer (DO), and when used to supplement active recruitment by staff either through door-to-door contact (IVS, EHBR) or providers (PBR).

Tailored activities conducted for 1–2 years before the start of recruitment were associated with substantial percentages of women who reported that they had heard about the NCS at initial screening. The DO group had the highest percentage of women who had heard, not surprising given that DO recruitment was dependent on self-referral. Kaar *et al*. [20] observed that women who said they had not heard were those whose first contact with the study was when they received the Pregnancy Screener in the mail. In addition to the DO group, the percentages of EHBR and PBR women who had heard were also substantially higher than the IVS, strongly suggesting that the more structured and extensive outreach and engagement in the ARS was most effective.

The NCS provides strong support for the importance of multifaceted outreach and engagement for successful recruitment. Across recruitment groups, the more sources women heard from, the higher the odds of consent. Depending on the recruitment strategy, different methods of outreach and engagement affected the odds of consenting differently. Word-of-mouth, hearing about the study from a healthcare provider or another person, increased the odds of consent across ARS groups. For 3 of the 4 recruitment types, those that emphasized self-referral (DO) or were household-based (IVS, EHBR), approximately one-third of the women had heard through word-of-mouth. In the PBR group, that percentage was much higher because, as would be expected, over half of the women had heard about the study from a healthcare provider. Interestingly, hearing from a provider did not increase the odds of consent in that group, but hearing from another person did.

Of the women who heard about the study, 20%–42% heard from the media—print, TV, or radio. Media outreach was much more extensive in the ARS than the IVS, with both national and local campaigns, and more widespread use of radio, TV, online and social media. Media outreach predicted consent in 2 of the 3 ARS groups, DO and EHBR. This finding is consistent with Maghera *et al*. [9] who found that passive recruitment using free and paid media had strong effects in predicting consent when compared with active contact from a healthcare provider. In an analysis of DO recruitment, Kaar *et al*. [20] observed that in locations with high NCS awareness (≥75% of women who had heard), higher percentages of women said that they had heard through the media. The impact of media outreach is likely underestimated here because many of the people who told others about the study (word-of-mouth) across recruitment groups quite likely heard about it through media in the first place. Manca *et al*. [10] found that nearly 3 quarters of the consented women in one of their pregnancy cohorts reported that word-of-mouth or media outreach was the primary recruitment method.

In the Vanguard Study, except for 3 PBR locations, all centers mailed advance letters to households. The letters were one of the most frequent ways that women reported that they had heard about the study. The literature regarding randomized clinical trials suggests that mass mailing may increase recruitment [21, 22]. However, in this study, advance mailings only predicted consent for PBR. It may be that advance mailings primed women to be more receptive when approached within a trusted setting where provider approval might be inferred, but not when women were being asked to self-refer or to make a commitment to a stranger approaching them in their homes. Indeed, Promislow *et al*. [11] reported that only 5% of their pregnancy cohort was recruited through mailings. And a systematic review of strategies to improve recruitment to clinical trials found no effect in 2 trials of advance letters or postcards as supporting methods for active recruitment [2].

Study results were mixed regarding demographics and whether women heard about the study. In general, non-Hispanic Whites were more likely than other ethnic/racial groups to have heard before being approached. However, there were 2 notable exceptions. In the EHBR group, which had the highest percentage of Hispanics, Hispanics were equally likely as non-Hispanic Whites. And in the DO group, African-Americans were twice as likely to have heard. Across all recruitment groups, women with a bachelor’s degrees or postgraduate education were more likely to have heard than women with a high school education. Our findings suggest that it is possible to tailor outreach and engagement to successfully reach ethnically and racially diverse samples, but emphasize the need to tailor approaches for diverse educational backgrounds.

This article has several limitations. First, there were significant interactions between all demographic variables and recruitment type and the demographic results should be viewed with caution. Differences between recruitment strategies could be due to differences in demographics. A comprehensive regression model containing all main and interaction effects would not be interpretable. Second, specific community outreach and engagement strategies were not quantified. Although all locations had similar stated expectations from NIH, different groups may have allocated significantly different levels of resources to outreach and engagement. Finally, the Vanguard Study was a feasibility study and the duration of recruitment varied across the locations and recruitment groups. For example, the PBR group had to negotiate access with providers before they could begin recruiting and in some locations, actual recruitment only lasted a few months. Other researchers have reported changes in patterns of cohort recruitment at 6 or 12 months [8, 11]. Despite this limitation, 1 strength of this study is that the final sample included nearly 10,000 study-eligible women.

## Conclusion

Outreach and engagement are often some of the most time-intensive and costly aspects of study implementation, especially for longitudinal studies. This study demonstrates that multifaceted, place-sensitive outreach and engagement facilitates successful passive and active recruitment of a prebirth cohort. Effective approaches must be based on community input and a strong working knowledge of the local community. Media outreach and strategies to encourage word-of-mouth appear to be critical to successful recruitment.

## References

[1] Galea S, Tracy M. Participation rates in epidemiologic studies. Annals of Epidemiology 2007; 17: 643–653. 17553702 10.1016/j.annepidem.2007.03.013

[2] Mapstone J, Elbourne D, Roberts IG. Strategies to improve recruitment to research studies. Cochrane Database of Systematic Reviews [Internet] 2007; 2: 1–21 [cited Apr 16, 2015]. (http://doi.wiley.com/10.1002/14651858.MR000013.pub3). 10.1002/14651858.MR000013.pub3 17443634

[3] Treweek S, et al Strategies to improve recruitment to randomised controlled trials. Cochrane Database of Systematic Reviews [Internet] 2010; 4: 1–109 [cited Apr 16, 2015]. (http://onlinelibrary.wiley.com/doi/10.1002/14651858.MR000013.pub5/full). 10.1002/14651858.MR000013.pub4 20091668

[4] Sapienza JN, et al Community engagement in epidemiological research. Ambulatory Pediatrics 2007; 7: 247–252. 17512886 10.1016/j.ambp.2007.01.004 PMC1978546

[5] Brenner BL, Manice MP. Community engagement in children’s environmental health research. Mount Sinai Journal of Medicine: A Journal of Translational and Personalized Medicine 2011; 78: 85–97. 10.1002/msj.20231 PMC3086533 21259265

[6] Golding J, Birmingham K. Enrolment and response rates in a longitudinal birth cohort. Paediatric and Perinatal Epidemiology 2009; 23: 73–85. 19490447 10.1111/j.1365-3016.2008.01001.x

[7] Barbara AI, et al Review of community-based research: assessing partnership approaches to improve public health. Annual Review of Public Health 1998; 19: 173–202. 10.1146/annurev.publhealth.19.1.173 9611617

[8] Webster GM, Teschke K, Janssen PA. Recruitment of healthy first-trimester pregnant women: lessons from the Chemicals, Health & Pregnancy Study (CHirP). Maternal and Child Health Journal 2011; 16: 430–438. 10.1007/s10995-010-0739-8 21210200

[9] Maghera A, et al You are how you recruit: a cohort and randomized controlled trial of recruitment strategies. BMC Medical Research Methodology 2014; 14: 111. 25260762 10.1186/1471-2288-14-111 PMC4190339

[10] Manca DP, et al The most effective strategy for recruiting a pregnancy cohort: a tale of two cities. BMC Pregnancy and Childbirth 2013; 13: 75. 23521869 10.1186/1471-2393-13-75 PMC3614477

[11] Promislow JHE, et al Recruitment for a community-based study of early pregnancy: the Right From The Start study. Paediatric and Perinatal Epidemiology 2004; 18: 143–152. 14996255 10.1111/j.1365-3016.2003.00546.x

[12] National Institutes of Health. Request for OMB clearance: recruitment substudy for the National Children’s Study, phases 1 and 2, part A only [Internet], 2011 [cited Aug 28, 2015]. (http://www.reginfo.gov/public/do/DownloadDocument?objectID=24135501 and http://www.reginfo.gov/public/do/PRAViewICR?ref_nbr=201104-0925-002).

[13] National Institute of Child Health and Human Development. *National Children’s Study* [Internet]. Bethesda, MD: National Institutes of Health, 2014 [cited Aug 28, 2015]. (http://www.nichd.nih.gov/research/NCS/Pages/default.aspx).

[14] Baker D, et al Recruitment of women in the National Children’s Study Initial Vanguard Study. American Journal of Epidemiology 2014; 179: 1366–1374. 24793429 10.1093/aje/kwu062 PMC4036215

[15] McGovern PM, et al The National Children’s Study: early recruitment outcomes using the direct outreach approach. Pediatrics 2016; 137(Suppl. 4): S231–S238. 27251869 10.1542/peds.2015-4410D PMC4878107

[16] Blaisdell LL, et al The National Children’s Study: recruitment outcomes using an enhanced household-based approach. Pediatrics 2016; 137(Suppl. 4): S219–S230. 27251868 10.1542/peds.2015-4410C PMC4878108

[17] Hale DE, et al The National Children’s Study: recruitment outcomes using the provider-based recruitment approach. Pediatrics 2016; 137(Suppl. 4): S239–S247. 27251870 10.1542/peds.2015-4410E PMC4878111

[18] National Children’s Study. *National Children’s Study Archive Study Description and Guide* [Internet], 2016 [cited Jan 30, 2017]. (https://ncsarchive.s-3.net/bioshare/).

[19] Slutsman J, Hirschfeld S. A federated model of IRB review for multisite studies: a report on the National Children’s Study federated IRB initiative. IRB: Ethics & Human Research 2014; 36: 1–6. 27390811

[20] Kaar JL, et al The experience of direct outreach recruitment in the National Children’s Study. Pediatrics 2016; 137(Suppl. 4): S258–S264. 27251872 10.1542/peds.2015-4410G PMC4878110

[21] Tooher RL, Middleton PF, Crowther CA. A thematic analysis of factors influencing recruitment to maternal and perinatal trials. BMC Pregnancy and Childbirth 2008; 8: 36. 18687110 10.1186/1471-2393-8-36 PMC2532678

[22] Hinshaw LB, Jackson SA, Chen MY. Direct mailing was a successful recruitment strategy for a lung-cancer screening trial. Journal of Clinical Epidemiology 2007; 60: 853–857. 17606183 10.1016/j.jclinepi.2006.11.005

